# Family sports interventions for the treatment of obesity in childhood: a meta-analysis

**DOI:** 10.1186/s41043-022-00317-7

**Published:** 2022-09-02

**Authors:** Lihong Yang, Chao Liang, Yaona Yu, Qian Xiao, Maomao Xi, Lixu Tang

**Affiliations:** 1 Present Address: School of Martial Arts, Wuhan Sports University, No. 461 Luoyu Rd. Wuchang District, Wuhan, 430079 Hubei Province China; 2 State Key Laboratory of Biocatalysis and Enzyme Engineering, School of Life Sciences, Hubei University, Wuhan, China; 3 Institute of Burns, Tongren Hospital of Wuhan University, Wuhan Third Hospital, Wuhan, Hubei China; 4 Beijing National Day School Uestc QUZHOU, Zhejiang, China

**Keywords:** Family sports, Children, Obesity, Meta-analysis

## Abstract

**Background:**

Obesity in children has become one of the key concerns of the World Health Organization, and the incidence of related non-communicable diseases is also rising. This study evaluates the effect of family sports participation on the treatment and prevention of obesity in children aged 0–14 years by systematic analysis.

**Method:**

A literature review from 2000 to 2020 was conducted. According to PRISMA-IPD (Preferred Reporting Items for MetaAnalyses of individual participant data) guidelines. The two researchers independently assessed the risk and bias of the articles, obtained a comprehensive, high-quality result, and extracted the data based on the Cochrane intervention system review manual. Randomized controlled trials (RCTs) were selected from the searches that used family sports interventions or family sports combined with dietary adjustments and behavioral habits change. Only studies targeting overweight or obese children aged 0–14 years were included.

**Results:**

The search resulted in a total of 16 studies. Across all 16 studies, there were a total of 1680 participants in the experimental groups and 1701 participants in the control groups. The results are as follows: body mass index (*BMI*) (SMD-RE = − 4.10, 95% CI (− 0.84 to 0.02), *Z* = 1.88, *p* = 0.06); *Body weight* (SMD-RE = − 0.77, 95% CI (− 1.53 to − 0.01), *Z* = 2.00, *p* = 0.05); *Waist circumference* (SMD-RE = − 0.45, 95% CI (− 1.36 to 0.47), *Z* = 0.96, *p* = 0.34); and *Body fat rate* (SMD-FE = − 0.06, 95% CI (− 0.22 to 0.11), *Z* = 0.69, *p* = 0.49). Hence, through family sports intervention among obese children, juvenile and obese body composition—BMI, body weight, waist circumference, and body fat rate—are all reduced. But only body weight was statistically significant.

**Conclusions:**

Compared with the samples without family sports, the weight of obese children participating in family sports decreased, but there were no significant differences in other relevant physical indicators. Follow-up research should examine large-scale clinical trials with family sports as a single factor intervention, which are needed to provide stronger evidence of the intervention effect. However, family activities can help obese children grow and develop by improving their exercise capacity, enhancing their lifestyles, and facilitating communication and relationships with their parents. In the future, long-term sports training plans for children with obesity should be implemented.

## Background

Over the last 40 years, childhood obesity has increased tenfold. The World Health Organization (WHO) predicts that the number of obese children will increase from 41 million in 2018 to a staggering 70 million by 2025, and the proportion of overweight or obese people is worrying [1]. Obese children (aged 0–14) are the research objects of pediatric obesity in the field of medicine [2]. Obesity has been linked to internal environment disorder, gene variation, organ mutation, iatrogenic conditions, viruses, environmental and behavioral characteristics, and performance genetics [3]. However, the main mechanisms underlying childhood obesity are the consumption of high-caloric food, remaining sedentary for extended periods of time, and nonparticipation in physical activity and exercise [4]. When caloric intake is higher than consumption, excess calories are stored in the body in the form of fat, which leads to the imbalance of energy metabolism that results in obesity [5].

Presently, pediatric obesity treatment mainly attempts to limit energy intake by changing a child’s lifestyle One of these lifestyle changes is engagement in family sports. Family sports are organized and selected by family members to satisfy their family's enjoyment of life and health needs through physical exercise activities, effectively correcting children's bad habits and cultivating participation in lifelong sports [6–10]. Family sports intervention is commonly used in clinical medical experiments for physical therapy aimed at certain diseases. Long-term family physical exercise can reduce the incidence of cardiovascular diseases and chronic diseases in children.

Families bear the basic social responsibility of raising and educating children. Family sports is a supplement to school sports and plays an irreplaceable role. The intervention object of family sports is not only for children, but also for parents [11]. Parents play a key role in enabling their children's fitness, as they are responsible for directly supervising and managing children's behavior. Parenting behavior (e.g., role model behavior for imitation by children; setting rules and boundaries; etc.) influences children's behavior and eating habits. Childhood behavior plays a significant role in determining lifelong preferences and healthy behaviors, so it is necessary to shape and form a good lifestyle as soon as possible [9, 12].

Parents or supervisors directly organize and enact physical activity plans to realize off-campus sports activities. School sports supervision and organization of children's physical activities are limited in time and space [7]. The implementation of school sports and community sports requires family sports to assist: The community provides sports facilities and logistical support [13], and relevant education departments should continue to provide children and their families with opportunities for sports and health education, improve their self-efficacy in participating in sports activities, and support the ability of families to organize sports activities independently [14]. The organic integration of family, school, and community sports cultivates engagement with sports resources inside and outside the school, creating a convenient environment for children to participate in physical activities.

In summary, the occurrence of obesity is closely related to lifestyle and behavior habits. Scientific family sports intervention is one of the most important methods for treating and intervening in children's obesity. However, there is no meta-analysis on children's obesity by family sports intervention, and it is impossible to get a clear result analysis.

## Methods

This paper uses a meta-analysis method to systematically evaluate the intervention effect of family sports participation (single-factor and multifactor assistance) on children's obesity through a meta-analysis. Specifically, we examine how family sports intervention affects four key outcome indicators of obesity: *body mass index (BMI), weight, waist circumference, and body fat rate*.

### Search strategy and study selection

Example keywords searches include “Family Sports,” “Children,” “Obesity,” and “Family Sports,” among others. The following databases were searched: China National Knowledge Infrastructure (CNKI), VIP, Scopus, WanFang, PubMed, Embase, SpringerLink, ScienceDirect, Google Scholar, Cochrane Library, and Web of Science. The time period during which we searched the literature was 2006–2020.

We use the Web of Science search as an example in Table 1 to illustrate our study’s search strategy. In the basic search, each subject word was searched separately from the free word, and then a combination search in the historical search was performed. The index system included Science Citation Index Expanded (SCI-E), Social Sciences Citation Index (SSCI), Arts and Humanities Citation Index (A&HCI), Conference Proceedings Citation Index—Science (CPCI-S), Conference Proceedings Citation Index—Social Sciences and Humanities (CPCI-SSH), Emerging Sources Citation Index (ESCI), Current Chemical Reactions—Expanded (CCR-E), and Index Chemicus (IC). The time span reflected all active years of research.

**Table 1:** Web of Science keyword search information statistics

Retractable	Search results	Keywords
#4	3915	#1AND#2AND#3
#3	1897272	Child* OR Adolescent
#2	58718	Family physical OR Family is sport OR Family physical education
#1	511120	Obesity OR Appetite depressants OR Body weight OR Lipectomy OR Bariatrics OR Skinfold thickness OR Anti-obesity

The results were exported and supplemented with literature tracing, manual search, and other methods to collect the text. A total of 4311 documents were retrieved. We used the EndNote X8 document management software to organize and review the documents. After reading the document titles, keywords, and abstracts, we excluded documents that were not related to the research intervention method, population, or research purpose of this topic, resulting in a sample of 125 papers. Finally, we excluded the data that did not sufficiently meet our statistical requirements for analysis. This resulted in a final sample of 16 papers.

Across all studies, the baseline of the experimental group and the control group was parallel and comparable, and all were clearly described.

### Inclusion/exclusion criteria

Next, we looked to incorporate the literature in the PICOS paradigm, which is reflected as follows:

- (P) Population. The research population is children (0–14 years old) with obesity. There are no restrictions on the gender and race of the study population.
- Intervention. The purpose of physical exercise through the family sports intervention is to meet a given family's needs for both enjoyment and, more importantly, physical health. Examples of family sports intervention include aerobic exercise, confrontation training, high-intensity interval training and continuous training, low-intensity large-volume exercise, etc.
- (C) Control. The experimental structure relies on the intervention of family sports participation or family sports combined with other interventions (diet adjustment, behavioral habits intervention). The control groups saw no interventions.
- (O) Outcomes. The outcome index of treatment effects includes one or more of the following four obesity indicators: BMI, weight, waist circumference, and body fat rate.
- (S) The experiments were designed as randomized controlled trials.

**Fig. 1:**
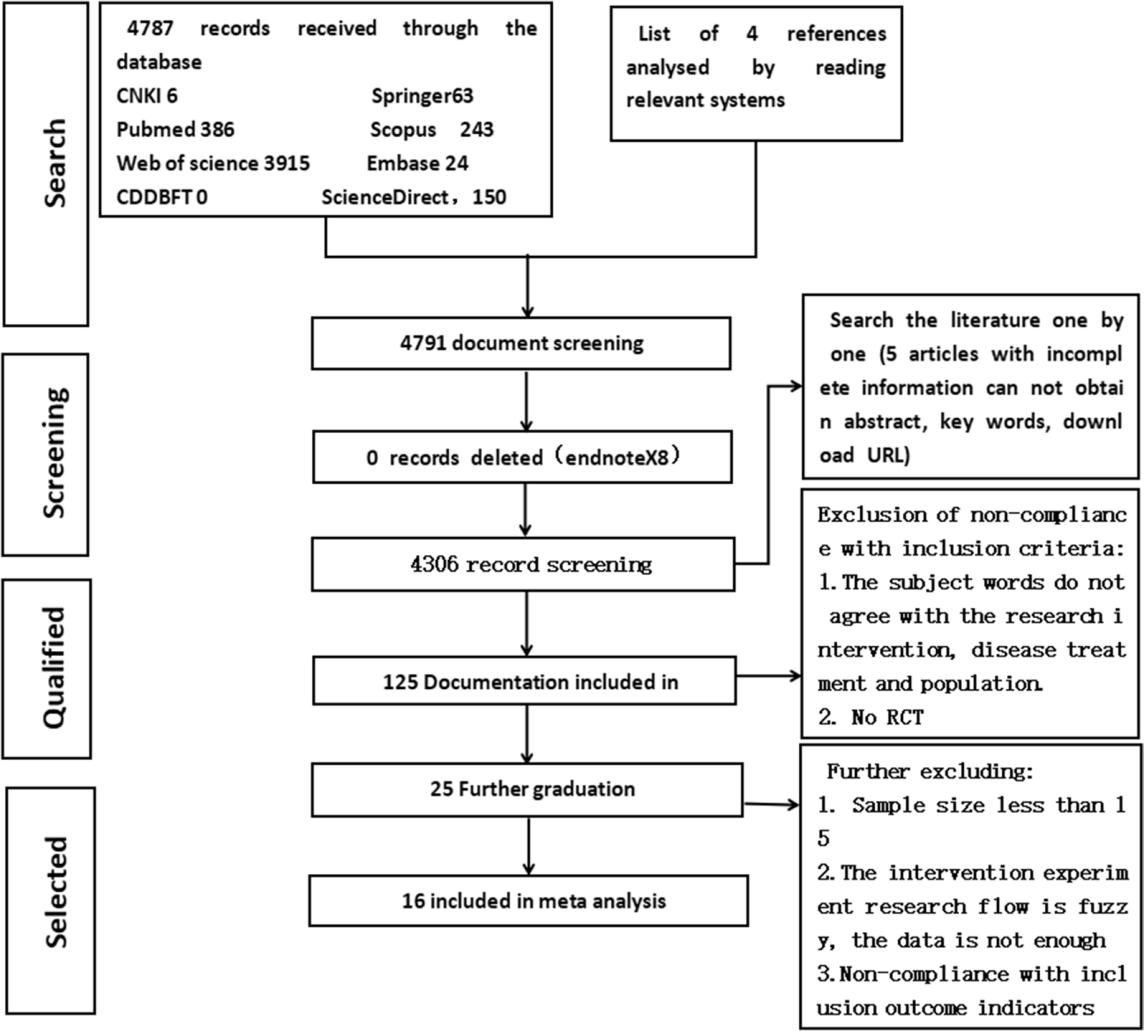
Flowchart of literature screening

The process of document selection and inclusion is shown in Fig. 1. EndNote X8 document management software was used to organize and review the documents, read the document titles, keywords, and abstracts, and exclude documents that were irrelevant to the research intervention method, population, or research purpose of this topic. The Chinese literature search was restricted to core journals, such as SCI, CSCI, and CSCD. Non-Chinese studies were assembled from SCI-E, SSCI, A&HCI, CPCI-S, CPCI-SSH, ESCI, and CCR-E.

Studies that emerged from the search results were excluded if they were not RCTs, had a sample size of less than 15, or whose research flow with regard to the intervention experiment was vague. Additionally, studies were excluded whose outcome indicators did not have one of the four indicators of BMI, weight, waist circumference, and body fat rate. There are no four indicators in the outcome indicators of the study. There are baseline data, lack of outcome data, and incomplete data in the research results.

### Data extraction and quality assessment

Data were extracted independently by two authors using a standardized data extraction form. The extracted data were entered into a specialized database and checked independently by the third author. The basic information extracted included author, year, sample size, intervention/controls details, and outcome variables (weight, waist circumference, BMI, and body fat rate).

The quality assessment of the literature needed to be assessed by two independent authors using the Cochrane Risk of Bias Assessment Form. Quality assessment was performed for each study included in this meta-analysis, and a third reviewer was required to perform the quality assessment if disagreement remained after the assessment by two authors.

The literature risk quality evaluation of the 16 studies includes:

- the selection of random methods;
- whether there was implemented allocation concealment;
- whether the participants and staff were blind;
- whether the results were assessed using a blind method;
- whether there were incomplete reporting outcomes;
- whether there were selective reporting and other deviations.

### Data synthesis and statistical analysis

Data statistics and meta-analysis were performed using RevMan 5 software, and effect scales were calculated by calculating weighted mean differences (WMD) or standardized mean differences (SMD) and 95% confidence intervals (CI) to assess the effectiveness of the home exercise intervention in each outcome indicator. Fixed-effects model tests were used in case of consistent outcome indicators or when the studies were homogeneous (*I*2 ≤ 50%, *p* > 0.1), and random-effects model tests were used in case of large heterogeneity in outcome indicators (*I*2 > 50%, *p* < 0.1).

The results of the analysis were interpreted using forest plots, and obese children were divided into two groups by child age classification criteria: preschool age 0–6 years and school age 7–14 years, and subgroup analysis was performed for each outcome indicator.

## Results

### Description of the included studies

The flowchart of studies’ selection is presented in Fig. 1. Our electronic search identifies 4787 studies (6 from CNKI, 386 from PubMed, 3915 from Web of Science, 63 from Springer, 243 from Scopus, 24 from Embase, and 150 from ScienceDirect). Of those studies, 1897 citations were retrieved after the exclusion of duplicates. Among them, 0 study was excluded in EndNote literature management software. Then, screening is done by two authors reading the title and abstract. Both were required to reach a consensus, and if disagreement still existed after discussion, the opinion of a third reviewer was solicited. Ultimately, 16 studies met all of the inclusion criteria and were eligible to be included in the final meta-analysis [15–30]. Tables 2 and 3 provide an overview.

**Table 2:** Basic information statistics included in the literature

Author, year	*N*	Intervention/controls details	Age	Intervention	Outcome variable (S)
Rong et al. 2007	140	Intervention group, *n* = 70 Control group, *n* = 70	3–6	Family sports, diet adjustment, behavior habits intervention	Weight
Zeng et al. 2013	70	Intervention group, *n* = 35 Control group, *n* = 35	3–6	Family sports, diet adjustment, behavior habits intervention	Waist circumference BMI
Luo et al. 2017	92	Intervention group, *n* = 42 Control group, *n* = 50	0–6	Family sports, diet adjustment,	BMI
Wang 2016	120	Intervention group, *n* = 60 Control group, *n* = 60	3–6	Family sports, diet adjustment, behavior habits intervention	Weight
Saelens et al. 2011	29	Intervention group, *n* = 15 Control group, *n* = 14	7–11	Family sports, diet adjustment	Weight, body fat rate BMI, waist circumference
Rodearmel et al. 2015	218	Intervention group, *n* = 116 Control group, *n* = 102	7–14	Family sports, diet adjustment	Weight, body fat rate BMI, waist circumference
Okely et al. 2010	102	Intervention group, *n* = 60 Control group, *n* = 42	5.5–9.9	Family sports, diet adjustment,	Weight, BMI, waist circumference
Ahmad et al. 2018	122	Intervention group, *n* = 64 Control group, *n* = 58	8–11	Family sports, diet adjustment, behavior habits intervention	Body fat rate
Abbey Alkon et al. 2014	552	Intervention group, *n* = 260 Control group, *n* = 292	3–5	Family sports, diet adjustment	BMI
Döring et al. 2016	1014	Intervention group, *n* = 458 Control group, *n* = 556	0–4	Family sports, diet adjustment, behavior habits intervention	BMI, waist circumference
Nyström et al. 2018	263	Intervention group, *n* = 133 Control group, *n* = 130	4.5	Family sports, diet adjustment	Weight
Sacher et al. 2010	116	Intervention group, *n* = 60 Control group, *n* = 56	8–12	Family sports, diet adjustment	Body fat rate BMI, waist circumference
Oliva et al. 2014	306	Intervention group, *n* = 168 Control group, *n* = 138	2–5	Family sports, diet adjustment	BMI
Jyu-Lin Chen et al. 2009	67	Intervention group, *n* = 35 Control group, *n* = 32	8–10	Family sports, diet adjustment	BMI
Amy et al. 2015	80	Intervention group, *n* = 59 Control group, *n* = 21	8–12	Family sports, diet adjustment	Waist circumference, BMI
Epstein et al.2000	90	Intervention group, *n* = 45 Control group, *n* = 45	8–12	Family sports, diet adjustment, behavior habits intervention	Weight, body fat rate

**Table 3:** Statistics of basic information of family sports

Aged 0–6 years				
Author	Frequency, times/week	Time (min)	Intensity	Program
Zeng et al.	≥ 5	> 20	Low to medium intensity	Aerobic exercise such as cycling, jogging, climbing stairs, walking, etc.
Luo Juanjuan et al.	No	No	No	Implementation according to sports training plan
Abbey et al.	7	60	Medium to high strength	Moving sports balls, tricycles
Gloria et al.	7	120	No request	Running, jumping, walking, playing ball, swimming, dancing, etc.
Christine et al.	7	> 60	Medium to high strength	Physical activity, mobile wear accelerometer for recording
Nora et al.	7	60	Medium to high strength	Physical activity, no specific sports regulations, the physical activity boundary is 3908 cpm, light physical activity of 820 to 3907 cpm
Rong Xiujuan et al.	7	60	Medium intensity	Walk, run, jump, kick, climb stairs
Wang Jian	7	Stop after completing the target task	Low to medium intensity	Walking, running, jumping, climbing stairs
**C**				
Saelens et al.	6	60–90	Medium to high strength	Wear an acceleration calculator to calculate physical activity
Rodearmel et al.	7	Stop after completing the target task	Low to medium intensity	Record with pedometer, walking more than 22,000 steps a day
Anthony et al.	Not explained	Not explained	Not explained	Physical activity skills
Norliza et al.	4	30	Medium to high strength	Sports skills training
Paul et al.	7	25	High strength	Sports activities, no specific sports regulations
Jyu-Lin Chen et al.	No	15	No request	Game-based sports activities, including non-competitive dance, brisk walking, and skipping
Amy et al.	5	60	No request	Physical exercise games/traditional sports, for example yoga, body pumps, spinning classes, and group sports games
Epstein et al.	7	≥ 30	Medium to high strength	16.1 or 32.2 km, 10 or 20 miles per week

**Fig. 2:**
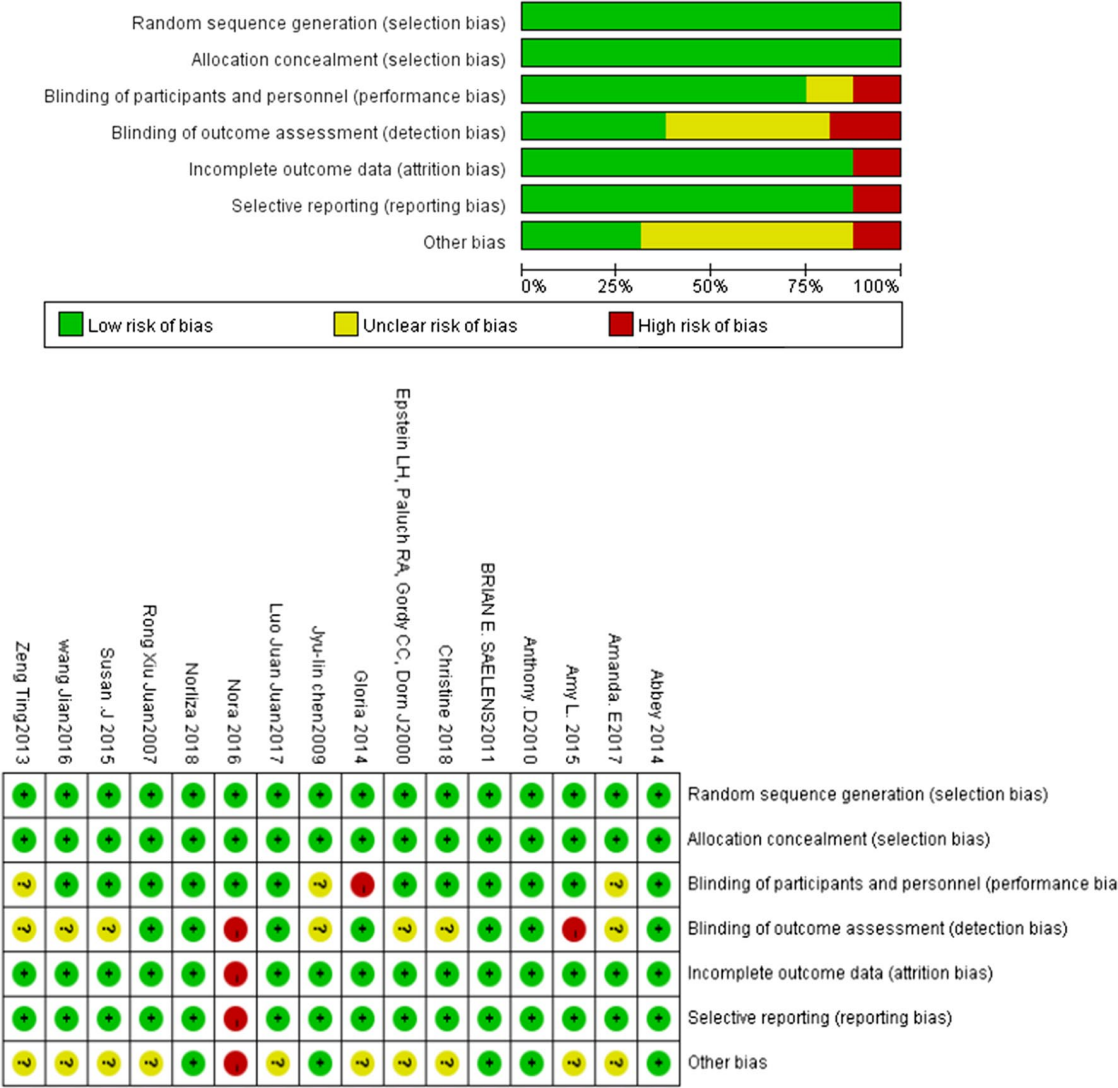
(1) Risk bias diagram. (2) Summary of risk bias

Figure 2(1) and (2) shows that the overall quality risk from the literature is low. The risk bias summary chart shows that two of the articles (Döring et al. 2016 and Sacher et al. 2010) have high-risk bias. The intervention measures are composed of different exercise forms and different exercise doses, so it is impossible to judge whether the above key factors have potential influencing effects. The above evidence is insufficient and threatens the high quality of results. Participants and staff guide the experiments, and the resulting evaluation is not blind. The results of the report are not comprehensive, and some reports are optional statements (Table 4).

**Table 4:** Family sports treatment strategies according to age and the degree obesity in children

Age group	Degree of obesity	Basic training	Intensive
Infant (< 2 years old)	Weight for height > 95th pc	Preventive precautions	Preventive precautions
Early Childhood (2–5 years)	BMI 5–84th pc	Preventive precautions	Preventive precautions
	BMI 85–94th pc risk $\emptyset$	Preventive precautions	Preventive precautions
	BMI 85–94th pc risk	Step 1	Step 2
	Present		
	BMI > 95th pc	Step 1	Step 3
Childhood (6–14 years)	BMI 5–84th pc	Preventive precautions	Preventive
	BMI 85–94th pc risk $\emptyset$	Preventive precautions	precautions
	BMI 85–94th pc risk	Step 1	Preventive
	Present		precautions
	BMI 95–99th pc	Step 1	Step 2
	BMI > 99th pc	Step 1	
		Parents participate in the selection of the second or third steps	Step 3
			Step 3
			Parents participate in the fourth step of choice

The Korean Pediatric Gastroenterology Hepatology and Nutrition Obesity Group classified obesity treatment and exercise strategies in four steps

We document that because of the close interaction between family members and research members, participants were aware of the measurement method, which directly precludes blind measurement. Seven of the documents did not explain whether they were blind to the staff, and thus there might be a high risk of bias. Only four documents had a low risk of bias. At present, a long-term randomized controlled study on family sports as the main single factor intervention for juvenile obesity is currently being run. The differences in the degree of intervention of family sports combined with diet and behavior intervention and the length of intervention (3–39 months) lead to differences in the effect of outcome indicators [31, 32].

### The impact of family sports intervention on children's BMI

**Fig. 3:**
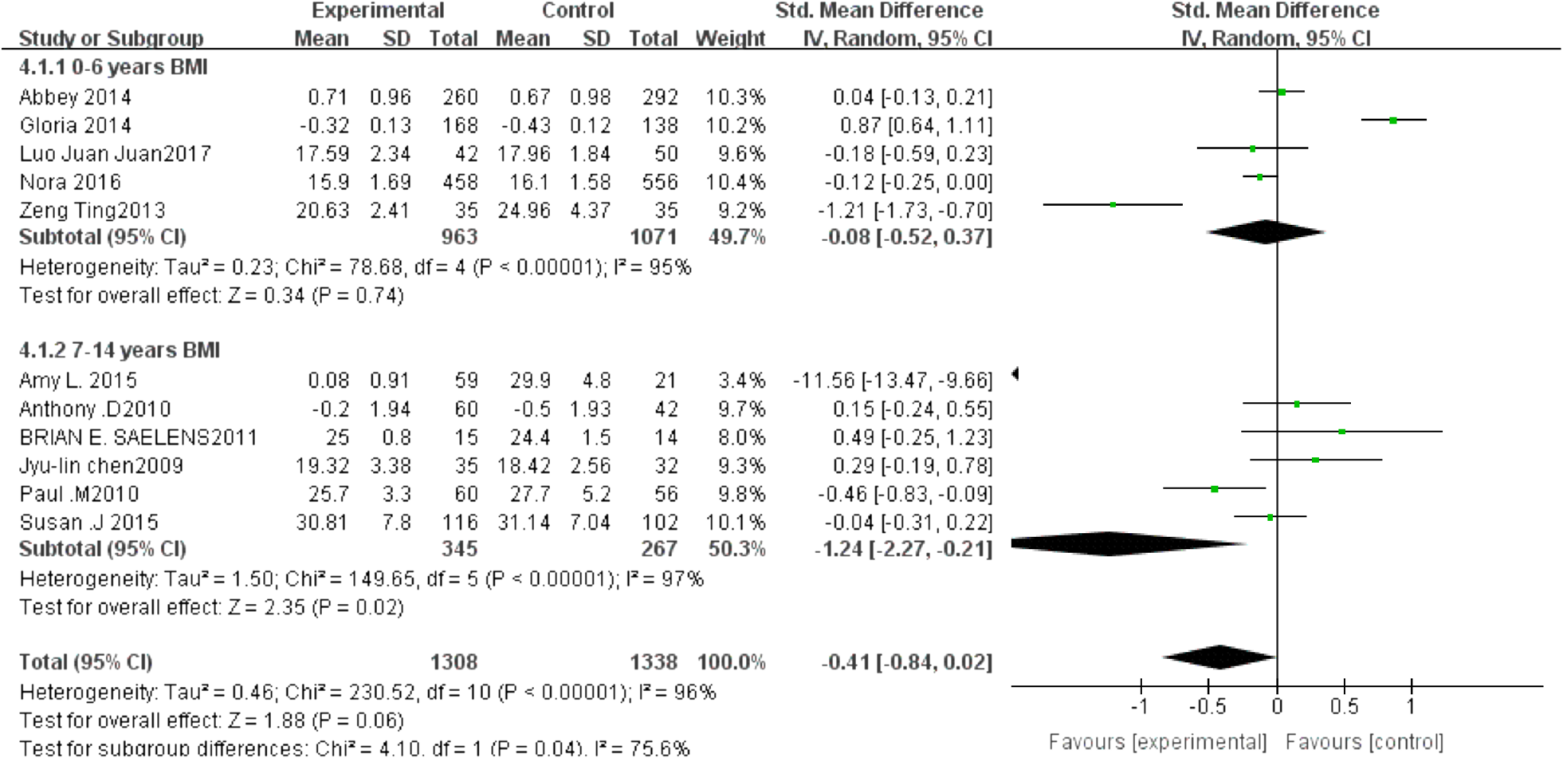
Outcome indicators BMI (0–6 years old, 7–14 years old) forest map of two subgroups

From the perspective of clinical analysis, the intervention population of each literature has a relatively large span. Ages are divided into two subgroups of 0–6 years and 7–14 years for data processing and analysis. As can be seen from the forest chart in Fig. 3, the participation of family sports has a significant impact on the BMI of obese children.

For the 11 articles included in this analysis, the outcome index BMI system shows *χ*2 = 0.46, df = 10, *p* < 0.00001, *I*2 > 50%. The heterogeneity of the included literature is relatively high, and it can be analyzed with a random-effects model. SMD-RE = − 0.41, 95% CI (− 0.84 to 0.02), *Z* = 1.88, *p* = 0.06, which is not statistically significant. Among them, there are five subgroups of 0–6 years old and six subgroups of 7–14 years old, respectively. There were 1308 experimental groups and 1337 control groups.

Meta-results of 11 literature subgroups show *χ*2 = 4.10, df = 1, *p* = 0.04, *I*2 = 75.6%, and moderate subgroups differences decreased less. We exclude each article piecemeal to check the sensitivity and observe the results of the effect size change. The research results show that the merged results are highly robust. The total statistics reflect the overall situation of the 11 studies, and the effect differences between the experimental group and the control group are higher.

**Fig. 4:**
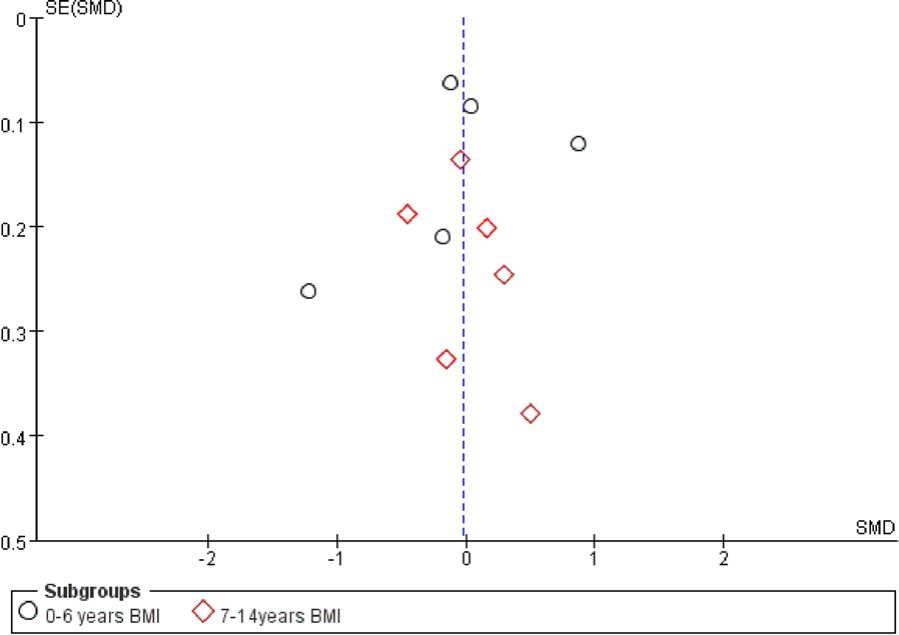
Funnel chart of two subgroups of outcome indicators BMI (0–6 years old, 7–14 years old)

The results show that the intervention of family sports can reduce the BMI of obese children. Figure 4 shows two subgroup funnel charts: The white dots and red dots are concentrated in the middle and top of the vertical axis of the graph, indicating that the sample size is large, indicating greater accuracy. Hence, it is closer to the true value, and the distribution of points is relatively concentrated. Specifically, we observe that the publication bias of the above literature is low.

### The effect of family sports intervention on children's weight

**Fig. 5:**
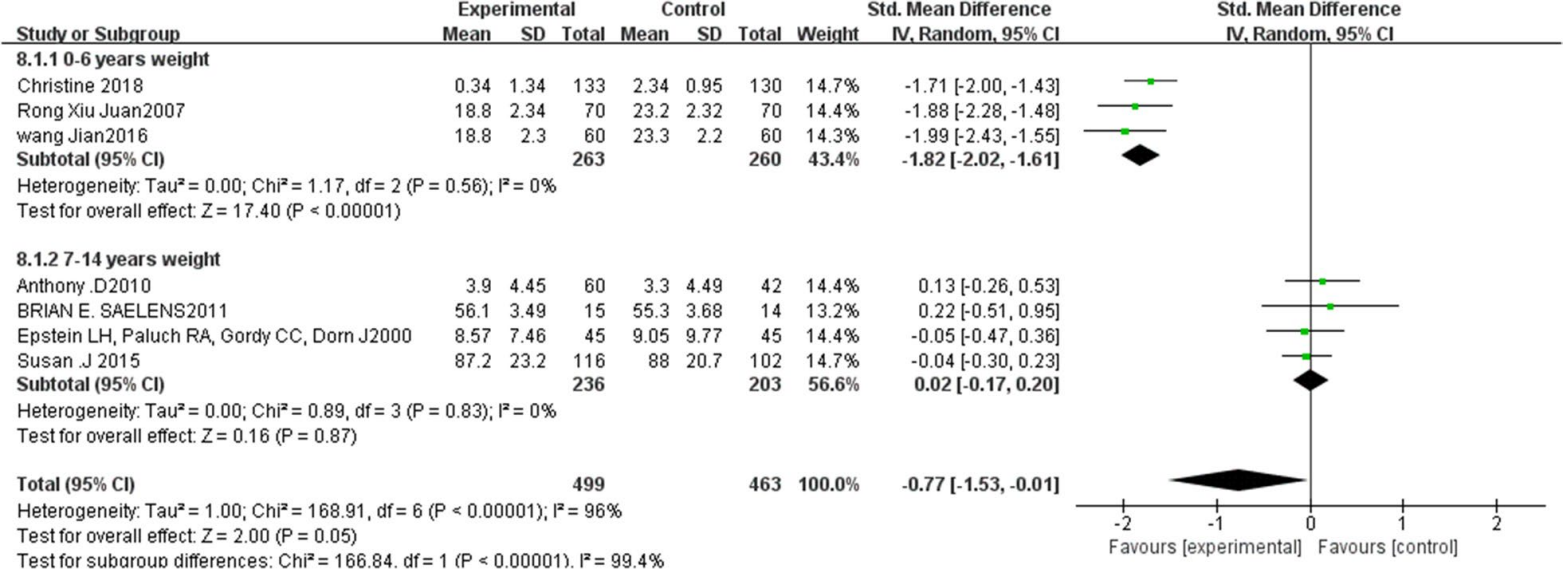
Outcome index weight (0–6 years old, 7–14 years old) forest map of two subgroups

The forest chart in Fig. 5 shows that family sports participation has a significant impact on the weight of obese children. A total of seven articles were included in this analysis. The outcome index weight system analysis shows: *χ*2 = 1.00, df = 6, *p* < 0.00001, *I*2 > 50%. The heterogeneity of the included literature is relatively high and can only be analyzed with a random-effects model.

The seven studies on the influence of body weight show SMD-RE = − 0.77, 95% CI (− 1.53 to − 0.01), *Z* = 2.00, *p* = 0.05, which has a statistical significance. The intervention group and the control group have significant differences before and after the intervention. After excluding each article one by one to check the sensitivity, we observe no change in the result of the effect size, indicating that there is no literature to be excluded.

Additional evaluation shows that the results of the literature data merger are more robust. Among them, there are three subgroups for 0–6-year-old children and four subgroups of 7–14-year-old children. There were 499 experimental groups and 463 control groups.

Results of the meta-analysis show that for 0–6-year-old children the heterogeneity is almost zero (*p* = 0.56, *I*2 = 0%), whereas the 7–14-year-old children group possesses greater heterogeneity (*p* = 0.83, *I*2 = 0%). The difference between the two subgroups and the total subgroup indicates the heterogeneity of the systematic analysis of the subgroup literature (*p* < 0.00001, *I*2 = 99.4%). The diamond shape falls in the experimental group, which shows the effect analysis of the two sub-combinations. The difference between the experimental group and control group clearly shows that participation in family sports has a significant effect on weight loss in obese children.

### The effect of family sports intervention on the waist circumference of children

**Fig. 6:**
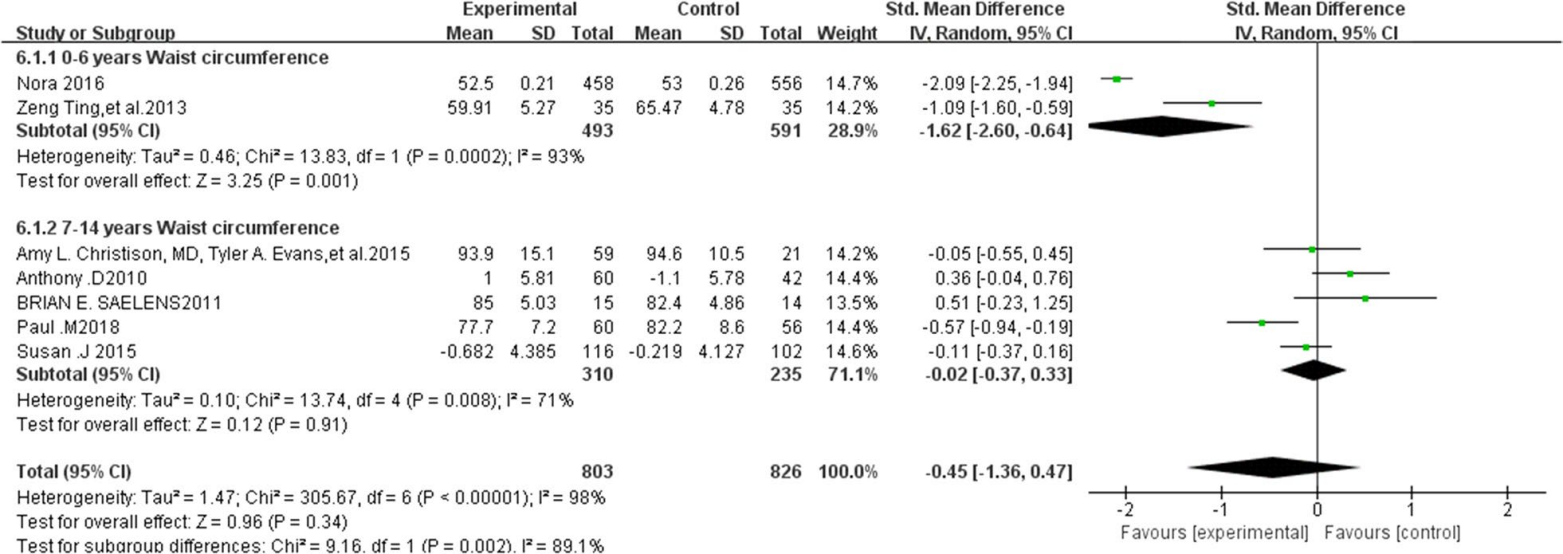
Two subgroup forest charts of outcomes indicators waist circumference (0–6 years old, 7–14 years old)

As can be seen from the forest map in Fig. 6, participation in family sports has a significant impact on the waist circumference of obese children. A total of seven articles were included in this analysis. The outcome index weight system analysis shows that the heterogeneity of the included literature is relatively high and can only be analyzed with a random-effects model (*χ*2 = 1.47, df = 6, *p* < 0.00001, *I*2 > 50%). Seven articles on the impact of waist circumference show that a significant effect is not obvious (SMD-RE = − 0.45, 95% CI (− 1.36 to 0.47), *Z* = 0.96, *p* = 0.34). The diamond on the forest map leans to the left in the associated forest chart (Fig. 6), which shows that there is a significant difference between the experimental group and the control group after the experiment. This difference is significant before and after the experiment. The experimental group had a significant reduction in waist circumference in children aged 0–6, but not in children aged 7–14 or overall.

By excluding each article one by one to check the sensitivity, we observe that the result of the effect size has no change. The consolidation result of the data from each article is robust. Among them, there are two subgroups of 0–6 years old and five subgroups of 7–14 years old. There were 803 experimental groups and 826 control groups. Results of the meta-analysis subgrouping indicate that the heterogeneity risk of systematic analysis of the subgroup literature is still high (*p* = 0.002, *I*^2^ = 89.1%).

The effect of the two sub-combinations is then analyzed. According to the total results, the difference between the experimental group and the control group is high, and the diamond on the forest map falls on the left side. The results show that participation in family sports has a significant effect on the waist circumference of obese children.

### The effect of family sports intervention on the body fat rate of children

**Fig. 7:**
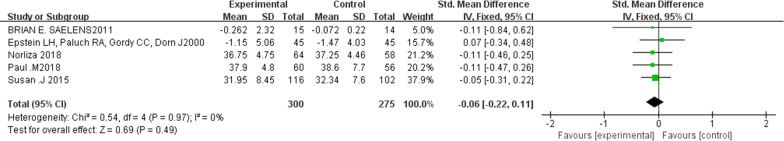
Forest map of outcome index body fat percentage

As can be seen from the forest map in Fig. 7, family sports participation has a significant impact on the weight of obese teenagers. A total of five articles were included. The age of the population belongs only to the 7–14-year-old children group; hence, no subgroup analysis of age was required. The experimental group included 300 children, and 275 participants were in the control group. Outcome indicators systemic analysis of body fat rate shows: *χ*^2^ = 0.54, df = 4, *p* = 0.97, *I*^2^ < 50%.

The homogeneity of the included literature is high, using a fixed-effects model analysis. Five articles on the impact on body weight show: SMD-FE = − 0.06, 95% CI (− 0.22 to 0.11), the total effect value *Z* = 0.69, *p* = 0.49. The statistical analysis shows no significance.

After excluding each article to check sensitivity, we find no change in the results of the effect size, indicating that there is no literature to be excluded, and the evaluation shows that the results of the literature data merger are relatively robust. The total effect of diamond was decreasing, but there was no statistical significance, indicating that there was no significant difference between the experimental group and the control group before and after the experiment. There was no significant effect on body fat percentage in obese children participating in family sports activities.

## Discussion

This project reflects the first comprehensive meta-analysis of the impact of family sports intervention on childhood obesity using a quantitative synthetic statistical method. We reviewed 16 RCT studies where the focal intervention method (family sports participation) and other intervention measures (diet adjustment and behavioral habit intervention) were used to treat and prevent obesity in children. The experimental intervention periods lasted at least 3 months.

We specifically select four common measurement indicators for meta-analysis across both age-groups [33]. Our analysis shows that the combined results of interventions on body weight are statistically significant, but the combined results of interventions on BMI, waist circumference, and body fat rate are not statistically significant. In particular, four common measures were selected for meta-analysis across two age-groups. Our analysis showed that the comprehensive effect of intervention on body weight was statistically significant, while the comprehensive effect on BMI, waist circumference, and body fat percentage was not. Only the comprehensive effect of body weight was statistically significant on the left side of the experimental group, indicating that the intervention of the experimental group could effectively reduce body weight. Our meta-analysis of increasing children's physical activities shows a different intervention effect as that of Brown, which studied the family-based intervention measures and found that children's physical activity level is increased through family participation [34, 35]. Additionally, Pamungkas studied family-based treatment and prevention interventions on children's obesity: Systematic review and meta-analysis disclose that family management can increase the frequency of physical activity and reduce sedentary time [36]. The possible reasons for this result are great differences in the level of evidence found in the research results, and the high risk of heterogeneity may be caused by the specific intervention populations and measures. These intervention populations have ethnic differences, the family economic level is inconsistent, and the age span of children is large. Moreover, the physiological development speed of men and women is different, which may affect obesity levels across gender[37, 38]. Most studies do not limit the spread of growth, social factors, and family class to the outcome indicators. Additionally, the 16 papers included in this study use a variety of intervention methods. Six studies use family sports, behavioral habits, and dietary adjustment for joint intervention, and 10 studies use only family sports intervention and dietary adjustment for intervention. System overview shows that the intervention effects of family sports intervention, dietary intervention, and behavioral habits alone are not as efficient as those of the combinations [39].

The research on the treatment and prevention of childhood obesity by British health improvement institutions mainly focuses on five topics: (a) family sports activities and interventions encourage and promote health; (b) family planning, taking parents as the main change element; (c) family behavior correction procedures; (d) behavior correction subjects are children; and (e) exercise treatment scheme [40].

Places family at the center of the intervention, as they are required to provide continuous exercise therapy, thereby cultivating an exemplary teaching environment for children in shaping their behavior habits. Family members are encouraged to supervise and manage the implementation of children's sports plans [41–43].

This study also offers a review of the design and recommendations for home exercise prescriptions for treating childhood obesity, as follows. The main purpose of family exercise therapy is to reduce the total fat content and visceral fat content of children and parents, increase the lean body weight, accelerate the consumption of excess energy in the body, maintain the balance of energy in the body, improve the level of resting metabolism, improve the level of lipid metabolism, and reduce metabolic and cardiovascular complications. When designing family exercise interventions, exercise training should reflect children’s growth and development rules, as well as age and gender differences, in order to appropriately enhance their physical health. For example, aerobic training, anti-group training, or combined exercise training are mainly used in home sports studies, and both types of exercise can effectively reduce total fat [44]. However, resistance exercise is more effective in increasing lean body weight and reducing the proportion of body fat [45].

Neral suggests principles to govern the use of family exercise therapy: (a) The principle of safe exercise should try to avoid sports injuries; (b) the principle of training plan arrangement varies from person to person; (c) the principle of gradual and orderly training load arrangement; (d) develop the principle of good behavior habits, reduce sedentary behavior, and increase physical activity; and (d) promoting overall health is the first principle [46].

Likewise, the Korean pediatric obesity group offers four strategies for exercise therapy: (a) Physical activity time should not exceed one hour. Young children participate in unstructured sports activities and older children participate in recreational sports activities; (b) exercise for one hour every day in an organized and planned way under parental supervision; (c) clarify the goal of physical activity in multidisciplinary obesity treatment, and make exercise plans for negative energy balance; and (d) appropriate exercise therapy strategies can be selected according to children of different ages and obesity degree.

Table 5 shows that the five factors considered in the design of family exercise prescription for obese children are exercise mode, exercise frequency, progressive load, exercise intensity, and duration. Family sports is characterized by varied and convenient daily physical activities wherein family members are encouraged to participate with children to increase physical activities, and children are the targets for intervention. Generally, obese children's primary exercise is arranged to walk for 10 min every 3–5 days, gradually increasing to exercise frequency every day. Exercise duration should be 60–80 min, with medium and high intensity (55%–90% maximum heart rate) as the main factor [47]. The monitoring and evaluation of exercise tasks provide records of goal tables and create an exercise log (space, duration, and prescribed training means of behavioral goals).

**Table 5:** Family sports plan for treating childhood obesity

Items and prescriptions	Aerobic exercise	Resistance movement	Physical activity	Stretching	Mixed motion
Exercise mode	Skateboard, racket, dance, basketball, football, volleyball, tennis, swimming, gymnastics, skipping, cycling, walking, hiking, skipping, climbing, running, kicking, and stair climbing	Rope climbing, tree climbing, rock climbing, push-ups, weightlifting, and boating	Children go to school on foot, play at rest, go hiking, walk with dogs, clean houses, park in farther places, use stairs instead of elevators or escalators	Yoga, dance, gymnastics	Competitive games, recreational skills learning (judo), dual tasks and mini sports games
Progressive load	Medium to high strength	High strength (50–70% MVC)	Low to medium strength	Medium to low intensity	Moderate to severe intensity
Motion frequency	> 3 times a week	2–3 times a week	Everyday	> 5 times a week	2 times a week
Exercise intensity	Medium and high strength	Medium and high strength	Medium and low strength	Low intensity	Moderate to severe intensity, the intensity is 65–75% of the maximum heart rate (HR)
Duration	20–90 min	2–3 min (8–20 times in each group, total times > 30)	> 20 min	10 min	60 min
Other	Wear a pedometer (measuring steps and stepping time)	Exercise major muscle groups	Not feel excessive fatigue, so as to avoid excessive	No clear exercise prescription	Portable HR detector

MVC, maximum voluntary contraction. The content of the family training schedule comes from the literature that meets the standards and the literature that adopts family sports/exercise intervention to treat and prevent childhood obesity

We also offer a number of suggestions for how to prescribe specific exercise regimens for children based on our research. These include: (a) developing appropriate exercise load according to children's obesity and children's physical fitness (especially patients with back, knee, and heel pain) to avoid joint injury; (b) paying attention to the foundation of children's early sports in order to cultivate healthy habits, interest in sports, and the concept of lifelong sports; and (c) paying attention to the growth and development of different ages and changes in human body composition [48–51].

For the treatment of childhood obesity, the main means are to increase physical activity and limit calories [52]. Specifically, obese children should focus on increasing lean body weight and reducing the proportion of body fat [53]. These children should strictly follow the sequence of sports training (preparation activities–intensive training–relaxation and stretching) and use social resources (environment, venue, facilities, media learning, etc.) [54, 55]. Diet therapy can help limit the total calorie intake of patients. Behavioral therapy can help patients improve sedentary behavior and lack of dyskinesia, and cultivate healthy eating and behavior habits [56]. The foundational elements of these behavior change skills include self-monitoring, goal setting, self-efficacy, problem-solving, recurrence prevention, and stimulation control.

## Conclusion

A meta-analysis of 16 studies showed that compared with the samples without family sports, the weight of obese children participating in family sports decreased, but there were no significant differences in other relevant physical indicators. However, the evidence is limited with high heterogeneity and bias in the literature, and no adverse events have been documented. Moreover, long-term regular interaction generates a healthy and close relationship between parents and children. In other words, family activities have a positive impact on children's growth, and intervention measures based on family sports can prevent children's obesity and act as an integral part of weight loss. Additionally, it is necessary to further study the efficiency of different forms of family sports intervention measures and avoid possible adverse events. In order to find more efficient treatment and prevention of childhood obesity, it is necessary to constantly practice and innovate the methods and means of family sports. Follow-up research should examine large-scale clinical trials with family sports as a single factor intervention, which is needed to provide stronger evidence of the intervention effect.

In the future, long-term sports training plans for children with obesity should be implemented. Relevant government departments and education departments should provide sports professional knowledge, behavioral intervention guidance, and continuous education support to instill knowledge of nutrition and empirically backed exercise regimens for children in different obesity treatment periods.

## Data Availability

The data that support the fundings of this study are available from the corresponding author upon reasonable request.

## Abbreviations

BMI: Body mass index
RCTs: Randomized controlled trials
CNKI: China National Knowledge Infrastructure
SCI-E: Science Citation Index—Expanded
SSCI: Social Sciences Citation Index
A&HCI: Arts and Humanities Citation Index
CPCI-S: Conference Proceedings Citation Index—Science
CPCI-SSH: Conference Proceedings Citation Index—Social Sciences and Humanities
ESCI: Emerging Sources Citation Index
CCR-E: Current Chemical Reactions—Expanded
IC: Index Chemicus
WHO: World Health Organization
SMD: STD mean difference
CI: Confidence interval
*Z*: *Z*-Score
SMD-RE: STD mean difference—random-effects models
SMD-FE: STD mean difference—fixed-effects models
WMD: Weighted mean differences
etc.: Et cetera
